# Channel Attention for Fire and Smoke Detection: Impact of Augmentation, Color Spaces, and Adversarial Attacks

**DOI:** 10.3390/s25041140

**Published:** 2025-02-13

**Authors:** Usama Ejaz, Muhammad Ali Hamza, Hyun-chul Kim

**Affiliations:** Department of Software, Sangmyung University, Cheonan 31066, Republic of Korea; usamaijaz404@gmail.com (U.E.); alihamzaiub13@gmail.com (M.A.H.)

**Keywords:** fire detection, deep learning, attention mechanisms, adversarial attacks, interpretability

## Abstract

The prevalence of wildfires presents significant challenges for fire detection systems, particularly in differentiating fire from complex backgrounds and maintaining detection reliability under diverse environmental conditions. It is crucial to address these challenges for developing sustainable and effective fire detection systems. In this paper: (i) we introduce a channel-wise attention-based architecture, achieving 95% accuracy and demonstrating an improved focus on flame-specific features critical for distinguishing fire in complex backgrounds. Through ablation studies, we demonstrate that our channel-wise attention mechanism provides a significant 3–5% improvement in accuracy over the baseline and state-of-the-art fire detection models; (ii) evaluate the impact of augmentation on fire detection, demonstrating improved performance across varied environmental conditions; (iii) comprehensive evaluation across color spaces including RGB, Grayscale, HSV, and YCbCr to analyze detection reliability; and (iv) assessment of model vulnerabilities where Fast Gradient Sign Method (FGSM) perturbations significantly impact performance, reducing accuracy to 41%. Using Local Interpretable Model-Agnostic Explanations (LIME) visualization techniques, we provide insights into model decision-making processes across both standard and adversarial conditions, highlighting important considerations for fire detection applications.

## 1. Introduction

The rapid rise in wildfire occurrences globally presents a significant environmental challenge, threatening ecosystems, infrastructure, and human life [1]. Data from leading national wildfire monitoring organizations reveal that wildfire impact has intensified by nearly threefold over the past ten years [2]. In 2022, the United States alone recorded 66,255 wildfires, affecting approximately 7.5 million acres of land [3]. Forests, comprising over 30% of Earth’s terrestrial area [3], are particularly susceptible to these fires, resulting in severe habitat destruction, biodiversity loss, and considerable declines in air quality [4,5]. This escalation, fueled by climate change and increased human activity, highlights the urgent demand for innovative fire detection systems [6]. The recent January 2025 wildfires in Los Angeles, exacerbated by severe drought and strong Santa Ana winds, further emphasized this need through widespread destruction [7]. Traditional sensor-based detection systems, while useful in localized areas, often struggle with real-time detection in remote and expansive environments, making them less effective for widespread fire monitoring [8]. Given this need, image-based fire detection has emerged as a promising alternative, leveraging deep learning to detect flames with improved precision [9,10,11,12,13,14,15,16,17,18,19,20,21,22,23].

Convolutional neural networks (CNNs) such as VGG19 [24] and ResNet [25] have shown potential for capturing complex flame-specific features across varied environmental conditions, advancing beyond traditional color and motion-based methods [26,27]. However, despite their effectiveness, CNNs frequently struggle with distinguishing flames from visually similar elements, especially in high-noise or dynamic environments, leading to inconsistencies in classification accuracy [26]. This limitation highlights the importance of enhancing feature specificity and reducing model reliance on background details for robust, real-world fire detection.

Moreover, fire detection systems can face vulnerabilities to adversarial perturbations, where minor input modifications can compromise model predictions—posing challenges in safety-critical applications like surveillance and environmental monitoring [28]. Adversarial training, including methods like Fast Gradient Sign Method (FGSM), has shown promise in fields like facial recognition for reinforcing model resilience [29]. However, adversarial robustness remains underexplored in fire detection, despite its potential to significantly improve reliability under varied conditions.

Data scarcity further complicates fire detection, as available datasets often lack diversity across environmental scenarios [30]. Data augmentation methods, such as rotation, scaling, and noise injection, help diversify samples and enable models to adapt better to new conditions. Effective augmentation is essential for enhancing model sensitivity and resilience, particularly in applications with limited labeled data.

To address the critical challenges in fire detection within high-noise, dynamic environments, we present a channel-wise attention-based architecture that emphasizes flame-specific features, with evaluations on adversarial effects and interpretability. Our approach applies structured data augmentation to broaden the training sample diversity, supporting consistent performance across varied fire scenarios. This model addresses specific limitations in traditional methods, providing a practical solution for complex real-world fire detection. The main contributions of this paper are:

- We propose an attention-based model by introducing channel-wise attention modules within the MobileNetV2 architecture, enabling the model to selectively focus on flame-relevant features while filtering out background noise. Through ablation studies, we validate that our channel-wise attention mechanism provides a 3% improvement in accuracy over the baseline architecture, achieving 95% accuracy on fire and smoke detection with precision scores of 96% and 97% on fire and smoke images respectively. Our proposed model improves the sensitivity and reduces false positives, particularly in high-noise environments where traditional CNN models often struggle.
- To address the limitations of fire detection datasets, we apply structured data augmentation, including rotation, scaling, flipping, and noise injection. Our proposed model trained with augmented images achieved 97% accuracy on fire and smoke detection. These augmentations increase sample diversity, enabling the model to generalize more effectively across varied environmental conditions and enhancing its adaptability to real-world fire scenarios. Moreover, we also perform evaluation across RGB, Grayscale, HSV, and YCbCr color spaces to explore the impact of color space on fire detection. Our results reveal RGB as the optimal choice, providing the highest accuracy of 95% due to its comprehensive color information crucial for distinguishing flame characteristics.
- Our proposed architecture outperforms state-of-the-art pre-trained models [31], including VGG16 (78%) [24], VGG19 (80%) [24], MobileNetV2 (92%) [19], EfficientNetB7 (82%) [32], ResNet50V2 (92%) [25], Xception (91%) [14], DenseNet121 (92%) [33], and InceptionV3 (92%) [34], as well as recent fire detection models like FireXplainer (92%) [17], FireXplainNet (90%) [35], and FireDetXplainer (91%) [36], achieving 95% accuracy and demonstrating a 3–5% improvement in detection performance across precision, recall, and F1-score metrics, underscoring its effectiveness for complex fire detection tasks.
- We provide interpretable insights into model decision-making across both standard and adversarial conditions, leveraging LIME visualization techniques. These visualizations not only explain model predictions by highlighting flame-relevant areas but also reveal how adversarial perturbations affect feature attention, demonstrating shifts in model focus that lead to misclassification. This analysis provides crucial understanding of model behavior under different operational conditions. We analyze model vulnerabilities to adversarial attacks using Fast Gradient Sign Method (FGSM) [28], revealing significant performance degradation under perturbations. Using LIME, we demonstrate how subtle input modifications lead to shifts in the model’s focus, substantially affecting classification reliability and providing insights into the vulnerabilities of deep learning models in fire detection systems.

The structure of this paper is as follows: Section 2 reviews existing deep learning-based approaches for fire and smoke detection. Section 3 presents our proposed approach, including dataset characteristics, preprocessing techniques, channel-wise attention-augmented MobileNetV2 architecture, experimental configuration, and evaluation framework. Section 4 evaluates model performance across baseline pre-trained models and state-of-the-art models, examining the impact of data augmentation, different color spaces, adversarial attacks, and LIME-based interpretability analysis. Section 5 examines the implications of experimental findings, model capabilities, and technical limitations. Section 6 concludes the study and outlines directions for future research.

## 2. Related Work

Fire detection has evolved significantly with the application of machine learning and deep learning, especially through image processing techniques [9,10,11,12,13,14,15,16,17,18,19,20,21,22,23]. Traditional fire detection methods based on color and motion analysis were early approaches in this field but often failed under varying lighting conditions, leading to high rates of false positives [10]. These early methods, while foundational, could not reliably handle dynamic environments where background changes impact detection accuracy. With the advancement of deep learning, convolutional neural networks (CNNs), such as VGG and ResNet, have become prevalent in fire detection research [14,17,19,26,27,33,37]. These models capture complex visual patterns specific to flames, improving the precision and recall of detection systems in diverse environments. However, despite their success in classifying fire and non-fire images, these models often lack the nuanced focus necessary to isolate flame-specific features, leading to inconsistencies in complex scenes where background elements interfere with model predictions [26].

Khan et al. [38] proposed a Cross-Module Attention Network (CANet) that combines squeezing and multi-scale feature selection to improve accuracy and computational efficiency in resource-constrained environments. Dilshad et al. [39] introduced the Optimized Fire Attention Network (OFAN), which leverages attention modules and lightweight architectures for real-time IoT-based fire detection. Yar et al. [40] presented a modified YOLOv5 architecture optimized for detecting small and large fire regions, addressing challenges in urban and indoor settings. Khan et al. [41] developed MAFire-Net, a multi-attention framework that enhances spatial and channel feature discrimination for accurate fire detection in complex scenarios. Additionally, Yar et al. [42] proposed an attention-based CNN model tailored for adverse weather conditions, incorporating data augmentation techniques to improve performance under foggy and low-light environments. Dilshad et al. [16] proposed E-FireNet, a VGG-inspired model fine-tuned for deployment on resource-constrained platforms like drones, ensuring robust performance in surveillance applications. Khan et al. [43] introduced a large-scale fire dataset encompassing diverse and challenging real-world scenarios, providing a broader benchmark for evaluating deep learning-based fire detection models.

FireDetXplainer, a MobileNetV3-based model using Grad-CAM and LIME, is introduced to improve interpretability in fire detection, achieving high accuracy while enhancing transparency in model decisions [36]. Additionally, FireXplainNet, a CNN-based architecture optimized for wildfire detection, utilized LIME to achieve significant accuracy gains on the FLAME and Wildfire datasets [35]. Similarly, FireXplainer [17], a transfer learning-based model incorporating convolution blocks and Grad-CAM for interpretability, demonstrated specific improvements in precision, recall, and F1-score on wildfire detection dataset.

In recent years, attention mechanisms have been integrated into deep learning architectures to improve feature extraction specificity, enabling models to concentrate on key regions of interest [9,44]. For instance, attention layers in CNNs have shown effectiveness in medical imaging and object detection, enhancing classification performance by isolating task-relevant features and reducing background interference [9]. An attention-augmented VGG19 model, for example, demonstrated quantifiable improvements in sensitivity and specificity by prioritizing critical regions in images [9]. While some studies have introduced attention mechanisms for fire detection [42,45], further exploration is needed to refine the isolation of flame-specific features, particularly in high-noise, variable environments. This gap highlights the need for attention-based fire detection models that can accurately capture flame characteristics amidst complex backgrounds, a critical requirement for reliable fire detection in real-world scenarios.

Adversarial vulnerabilities present additional challenges in fire detection systems, as CNN models are susceptible to minor but structured perturbations that can lead to significant misclassification errors. Methods such as the Fast Gradient Sign Method (FGSM) and Projected Gradient Descent (PGD) expose these weaknesses, even when low-level perturbations are introduced, affecting the stability of CNNs in adversarial conditions [28]. Such vulnerabilities raise concerns regarding the reliability of CNNs in real-world fire detection, where environmental noise or targeted attacks can compromise model performance. In safety-critical applications such as facial recognition and surveillance, adversarial training with FGSM has been employed to reduce classification errors and improve model stability under adversarial conditions [29].

Building on advancements in attention mechanisms [9], data augmentation [30], and adversarial analysis [28,29], this study introduces a MobileNetV2 model enhanced with channel-wise attention mechanisms, specifically optimized for the detection of flame features in complex environments. The proposed model incorporates channel-wise attention to focus on flame-specific characteristics, effectively reducing false positive rates caused by background interference. The vulnerability of the model to adversarial perturbations is systematically evaluated using FGSM, providing insights into its stability under potential adversarial conditions. To address data limitations and enhance generalizability, structured data augmentation techniques—such as rotation, flipping, scaling, and noise injection—are employed to increase dataset diversity. Model interpretability is facilitated using LIME, offering transparent visualizations of the decision-making process, which is crucial for deploying fire detection models in real-world scenarios. This comprehensive approach aims to improve detection precision, reduce false positives, and ensure robustness across diverse fire scenarios.

## 3. Methodology

### 3.1. Overview

**Figure 1:**
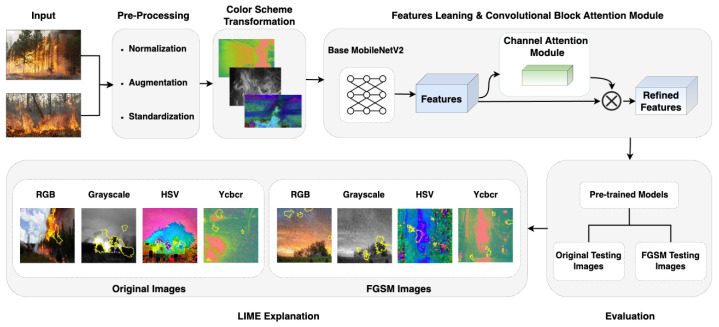
Workflow of our proposed methodology.

In this section, we present an overview of our proposed methodology for fire detection, as illustrated in the Figure 1. Our approach leverages the strengths of MobileNetV2 by incorporating pre-processing techniques, color transformation methods, and channel-wise attention mechanisms. The workflow begins with the input image undergoing pre-processing steps, including resizing, normalization, and noise reduction, to enhance image quality. The pre-processed image is then transformed using color schemes such as HSV and YCbCr to improve flame feature representation. Feature extraction is conducted using MobileNetV2, augmented with channel-wise attention modules to emphasize flame-relevant regions while suppressing background noise. Finally, the model is evaluated for robustness and accuracy using metrics such as precision, recall, and F1-score to ensure reliable performance across diverse fire scenarios.

The input images, consisting of fire, smoke, and neutral scenes, are first subjected to a series of pre-processing steps that include normalization, augmentation, and standardization. These pre-processing steps are crucial for enhancing the diversity of training data, mitigating overfitting, and ensuring that the model is trained under varied conditions to improve generalization. The pre-processed images are then transformed into different color schemes, including RGB, Grayscale, HSV, and YCbCr, to explore the impact of color representation on the model’s performance.

Feature extraction is performed using MobileNetV2 as the backbone, which is lightweight and well-suited for computational efficiency [17,36]. To further improve the model’s focus on relevant features, we incorporate a channel-wise attention-based module after feature extraction [46]. This module helps the model emphasize critical features that are essential for distinguishing between fire, smoke, and natural scenes, thereby enhancing classification accuracy.

The refined features are then passed to a classifier for training and evaluation. In the final stage, the model is evaluated using both original and adversarially perturbed images. We apply LIME [47] to visualize and interpret the key regions that influenced the model’s decision-making process under different conditions. By leveraging both original and adversarial testing images, our methodology not only focuses on achieving high accuracy but also highlights the vulnerabilities to adversarial attacks, providing critical insights into the model’s limitations in real-world fire detection scenarios.

### 3.2. Data Collection and Preprocessing

The dataset used in this study is the Fire-Smoke-Dataset, sourced from a publicly available repository [48] for fire and smoke detection, which provides a diverse collection of FIRE, SMOKE, and NON-FIRE images. It consists of 1000 high-quality images for each category: FIRE, SMOKE, and NON-FIRE images. This dataset has been widely recognized for its comprehensive nature and suitability for fire detection research [21,49], as it provides a balanced and diverse set of scenarios encompassing different levels of fire intensity, environmental conditions, and backgrounds.

To enhance the diversity of the training data and improve the model’s generalization, we applied a series of data augmentation techniques, including horizontal flipping, vertical flipping, random rotations, and scaling. The Fire-Smoke-Dataset used in this study consists of a total of 3000 high-quality images, with 1000 images per class (FIRE, SMOKE, and NON-FIRE). We split this dataset into training (2400 images, 800 per class), validation (300 images, 100 per class), and test (300 images, 100 per class) sets. These augmentation methods increased the total number of training images to 9600 (3200 per class), resulting in a 4-fold expansion of the training data. The validation and test sets remained unchanged to ensure a fair evaluation of the model’s performance.

The collected images underwent additional preprocessing tailored to explore two primary aspects of the classification task. First, we explored the impact of different color schemes on the multiclass fire detection problem by transforming the images into various color spaces, including RGB, Grayscale, HSV, and YCbCr. This enabled a systematic evaluation of the role of color information in distinguishing fire from natural images under different lighting and environmental conditions. All images are resized and normalized to a range of [0, 255] to ensure consistency in pixel value distribution across the dataset. These preprocessed images are then used to train and evaluate our enhanced proposed model with an attention mechanism, ensuring standardized input features for model training.

### 3.3. Proposed Model

Our proposed model is built upon MobileNetV2, a lightweight and computationally efficient architecture, which is highly suitable for real-time and edge applications [17,36]. While MobileNetV2 provides a foundation for feature extraction, its capabilities are further enhanced through the integration of a channel-wise attention module, designed to improve the model’s discriminative ability by emphasizing relevant features and suppressing redundant information. We inspired the block attention module from a previous study [46].

**Figure 2:**
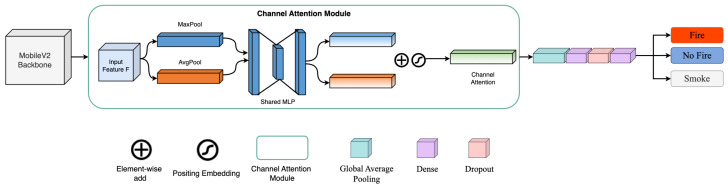
Our proposed architecture with Channel-wise attention modules.

The overall architecture of the proposed model is illustrated in Figure 2. The MobileNetV2 backbone extracts hierarchical features from input images, while the channel-wise attention mechanism is integrated after each inverted residual block to refine these features progressively. This design ensures that the attention module operates in tandem with the backbone, enabling the model to effectively capture discriminative patterns related to fire, smoke, and natural scenes under diverse conditions.

#### 3.3.1. Design of the Channel-Wise Attention Mechanism

The channel-wise attention mechanism selectively focuses on the most informative channels within the feature map, thereby enhancing the model’s ability to identify critical visual cues. The module operates as follows:

1. Input Feature Map: The input feature map $F\in R^{H\times W\times C}$, where *H*, *W*, and *C* represent the height, width, and number of channels, respectively, is fed into the attention module.
2. Parallel Pooling Operations: The feature map *F* is processed through two parallel branches:
  - Global Average Pooling (AvgPool): Computes the average activation for each channel, capturing global contextual information across spatial dimensions.
  - Global Max Pooling (MaxPool): Extracts the most salient features of each channel by selecting the maximum activation.
  These operations yield two descriptors, $f_{avg}\in R^{C}$ and $f_{max}\in R^{C}$, that encode complementary channel-wise information.
3. Shared Multi-Layer Perceptron (MLP): Both descriptors are passed through a shared MLP composed of two fully connected layers with ReLU activation. The shared design reduces parameter overhead while effectively learning channel-wise dependencies:
  $$f_{shared}=W_{2}\sigma \left(W_{1}f_{avg}\right)+W_{2}\sigma \left(W_{1}f_{max}\right),$$
  where $W_{1}$ and $W_{2}$ are learnable weight matrices, and σ denotes the ReLU activation function.
4. Element-Wise Addition and Attention Weight Calculation: The outputs of the two branches are aggregated via element-wise addition and passed through a sigmoid activation function to produce the final attention weights $\alpha \in R^{C}$:
  $$\alpha =\sigma (f_{shared}).$$
5. Feature Re-weighting: The attention weights α are applied to the original feature map *F* through channel-wise multiplication, generating the refined feature map $F_{out}\in R^{H\times W\times C}$:
  $$F_{out}=\alpha \cdot F.$$

This process ensures that the most relevant channels are amplified, while less significant ones are suppressed, resulting in improved feature representation for fire classification. The channel-wise attention module is seamlessly integrated with the MobileNetV2 backbone by placing it after each inverted residual block. This integration allows for progressive refinement of features at different stages of the network, complementing the hierarchical feature extraction process of MobileNetV2. The model achieves enhanced discriminative capability by focusing on channel-wise importance at multiple levels while maintaining computational efficiency.

#### 3.3.2. Connections Between Model Components

The proposed architecture is composed of the following key components:

- Feature Extraction and Refinement: The MobileNetV2 backbone serves as the primary feature extractor, while the channel-wise attention module refines the extracted features by selectively re-weighting channels based on their importance.
- Classification Head: The refined feature maps are passed through a series of fully connected layers, interspersed with dropout layers (dropout rate of 0.5) to prevent overfitting during training.
- Output Layer: The final fully connected layer employs a softmax activation function to produce class probabilities for three categories: FIRE, SMOKE, and NON-FIRE.

By combining the lightweight MobileNetV2 backbone with an efficient channel-wise attention mechanism, the proposed architecture achieves a balance between accuracy and computational cost. The integration of attention modules ensures that the model focuses on discriminative visual cues, enabling robust fire classification across diverse environmental conditions. This design is particularly advantageous for real-time and edge-based applications, where computational resources are constrained.

### 3.4. Experiment Setup

To evaluate the effectiveness of our proposed model for fire classification, we conducted extensive experiments on the Fire-Smoke-Dataset described earlier. The dataset consists of 1000 images each for FIRE, SMOKE, and NON-FIRE, split into training (2400 images, 800 per class), validation (300 images, 100 per class), and test (300 images, 100 per class) sets using an 80:10:10 ratio. We chose this dataset split inspired by other approaches for fire detection research [21,49], as it provides a balanced and diverse set of scenarios encompassing different levels of fire intensity, environmental conditions, and backgrounds. The impact of the data augmentation techniques applied to the training set, as detailed in Section 3.2, was to increase the total number of training images to 9600 (3200 per class), resulting in a 4-fold expansion of the training data.

Our proposed model is trained on these splits to assess its performance in distinguishing between fire, smoke, and natural scenes. The training process incorporates standard deep learning practices such as data shuffling, batch normalization, and early stopping to ensure convergence without overfitting. For training, we utilized the Adam optimizer with an initial learning rate of 0.001, and the learning rate was reduced by a factor of 0.1 if the validation loss plateaued for five consecutive epochs. The batch size was set to 32, and training was performed for up to 50 epochs with early stopping applied to halt training once validation performance stopped improving for 10 epochs. Hyperparameters were selected through a grid search approach, where we explored combinations of learning rates (0.0001, 0.001, 0.01), batch sizes (16, 32, 64), and dropout rates (0.2, 0.5) to optimize validation performance. The best configuration, based on validation accuracy and loss, was used for the final training. All experiments were conducted on a system equipped with two NVIDIA GeForce RTX 2080 Ti GPUs (each with 11 GB of memory), running on Driver Version 535.183.01 and CUDA Version 12.2. The training environment was implemented using PyTorch 1.7, leveraging the multi-GPU setup to accelerate model training.

In addition to evaluating our proposed model, we also fine-tuned several well-known pre-trained models to serve as benchmarks for comparison [31] and state-of-the-art fire detection models [17,35,36]. These models included VGG16 [24], VGG19 [24], MobileNetV2 [19], EfficientNetB7 [32], ResNet50V2 [25], Xception [14], DenseNet121 [33], and InceptionV3 [34]. By fine-tuning these established architectures, we aimed to provide a comprehensive comparison to highlight the strengths of our proposed model. Each model is fine-tuned using the same training and validation splits, allowing for a consistent evaluation across different architectures.

Furthermore, we conducted experiments to explore the impact of different color schemes on the classification performance of our proposed model. Specifically, we experimented with various color spaces, including RGB, Grayscale, HSV, and YCbCr, and channel-specific schemes such as Red, Green, and Blue, to gain insights into how color information influences fire detection. Moreover, we assessed the impact of data augmentation on model performance by applying techniques such as random rotations, flips, and scaling to the training images. The goal of these experiments is to evaluate whether increased data diversity can improve the generalization capabilities of our proposed model.

### 3.5. Evaluation Metrics

In the context of multi-class fire and smoke detection, evaluating the performance of our proposed model requires a thorough analysis using multiple metrics that offer insights into different aspects of model behavior. Given the complexity of classifying images into FIRE, SMOKE, and NON-FIRE categories, we employ accuracy, precision, recall, F1-score, and the macro-averaged versions of these metrics to comprehensively assess the model’s performance. This multifaceted approach ensures that both the correct identification of each class and the minimization of classification errors are addressed, making the evaluation process rigorous and informative.

Accuracy is a fundamental metric that represents the proportion of correctly classified images across all three classes. Specifically, it is calculated as the ratio of correctly classified instances (i.e., the sum of true positives for each class) to the total number of images. While accuracy provides a general overview of the model’s performance, it may be limited in its ability to convey class-wise effectiveness, particularly in cases where the dataset is imbalanced across the three categories. The formula for accuracy is given in Equation (1):

(1) $$Accuracy=\frac{TP_{fire}+TP_{smoke}+TP_{non-fire}}{Total\;Number\;of\;Images}$$

Precision, in a multi-class setting, measures the ability of the model to correctly identify positive predictions for each class while minimizing false positives. For each class (FIRE, SMOKE, and NON-FIRE), precision is defined as the ratio of true positive predictions to the sum of true positive and false positive predictions for that class. High precision across all classes is essential to reduce false alarms, particularly for the FIRE category, where false positives can lead to unnecessary disruptions. The macro-averaged precision is calculated by taking the mean of the precision values across all classes, as shown in Equation (2):

(2) $$Precision_{macro}=\frac{1}{3}\sum_{c\in \{fire,smoke,non-fire\}}\frac{TP_{c}}{TP_{c}+FP_{c}}$$

Recall, or sensitivity, is a crucial metric for assessing the model’s ability to correctly identify all positive cases for each class. In our multi-class fire detection problem, recall is calculated for each class as the ratio of true positive predictions to the sum of true positives and false negatives for that class. High recall is particularly important for the FIRE class to minimize the risk of undetected fires. The macro-averaged recall provides an overall indication of the model’s sensitivity across all classes, as given in Equation (3):

(3) $$Recall_{macro}=\frac{1}{3}\sum_{c\in \{fire,smoke,non-fire\}}\frac{TP_{c}}{TP_{c}+FN_{c}}$$

The F1-score, which is the harmonic mean of precision and recall, is used to balance the trade-off between these two metrics for each class. In a multi-class setting, the F1-score for each class is calculated individually, and the macro-averaged F1 score is obtained by averaging the F1-score across all classes. This metric is particularly useful in scenarios where both false positives and false negatives carry significant implications, such as avoiding false alarms while ensuring that fires are detected reliably. The macro-averaged F1-score is provided in Equation (4):

(4) $$F1_{macro}=\frac{1}{3}\sum_{c\in \{fire,smoke,non-fire\}}2\cdot \frac{Precision_{c}\cdot Recall_{c}}{Precision_{c}+Recall_{c}}$$

By employing these metrics, the evaluation of our proposed model becomes comprehensive, ensuring that we assess not only the overall accuracy but also the class-wise precision, recall, and F1-score. This holistic approach allows us to determine the strengths and limitations of the model across all relevant classes, ensuring its reliability and effectiveness in real-world fire detection scenarios.

### 3.6. Evaluation Methods

To evaluate the effectiveness of our proposed model, we conducted experiments across various color schemes, pretrained models, and model interpretability methods, aiming for a comprehensive understanding of model behavior in fire detection scenarios.

We trained and tested the model using multiple color representations, including multi-channel color spaces (RGB, HSV, YCbCr) and single-channel options (Grayscale, individual Red, Green, and Blue channels). The purpose of this exploration is to determine the impact of color information on fire detection performance. For example, RGB retains all primary color channels, preserving both color and intensity variations, whereas Grayscale removes color information entirely, emphasizing intensity alone. The HSV color scheme, on the other hand, focuses on hue and saturation variations, which can aid in distinguishing flames from other elements. By systematically testing these different representations, we sought to identify the most informative color scheme for effective fire detection. The RGB scheme ultimately provided the most comprehensive information for the model, resulting in superior performance compared to other schemes.

To further validate our model’s effectiveness, we compared its performance against several established pretrained models, including VGG16 [24], VGG19 [24], MobileNetV2 [19], EfficientNetB7 [32], ResNet50V2 [25], Xception [14], DenseNet121 [33], and InceptionV3 [34] and state-of-the-art fire detection models [17,35,36]. These models are fine-tuned using the best-performing color scheme (RGB) to ensure consistency in evaluation conditions. By using identical training and validation datasets, we ensured a fair comparison of the classification capabilities of each model. This evaluation revealed the strengths of our proposed model, highlighting its robustness and superior accuracy relative to these established architectures, particularly in scenarios involving complex backgrounds.

To enhance transparency in model predictions, we employed LIME [47] to analyze feature importance and identify critical regions that influenced classification. LIME is utilized to understand the key features that drove the model’s decision-making, both for original and adversarial images. By perturbing input features systematically, LIME provided insights into how the model localized flame-specific regions effectively, especially in RGB color space. This visual analysis revealed that the RGB scheme produced the clearest distinction between fire and non-fire areas, ensuring high interpretability and reliability in predictions.

The combination of color scheme evaluation, comparison with pretrained models, and LIME-based interpretability allowed us to thoroughly assess the model’s capabilities and its ability to focus on relevant fire features, even in challenging conditions. This holistic approach aims to ensure that our model is not only accurate but also can interpretable and adaptable to real-world fire detection applications.

## 4. Results

### 4.1. Comparison with Baseline Methods

To evaluate the effectiveness of our proposed model, we compare it against well-established state-of-the-art models [31] using the RGB color scheme, which is identified as the most effective in our earlier evaluations [31]. The baseline models included commonly used architectures such as VGG16 [24], VGG19 [24], MobileNetV2 [19], EfficientNetB7 [32], ResNet50V2 [25], Xception [14], DenseNet121 [33], and InceptionV3 [34]. All models are trained and tested on the same Fire-Smoke-Dataset with identical conditions to ensure a fair comparison.

As presented in Table 1, our proposed model demonstrated superior performance across all evaluation metrics when compared to these baseline methods. Specifically, our model achieved a precision of 96%, 0.98%, and 97% for the FIRE, SMOKE, and NON-FIRE classes, respectively, with an overall macro-averaged precision of 97%. The recall and F1-score similarly reflected strong performance, with the model achieving a recall of 96% for Fire and Smoke, and 99% for Neutral, resulting in a macro-averaged recall of 97%. The overall accuracy of our proposed model reached 97%, indicating a consistent and reliable performance across all classes.

The baseline models, while effective, are outperformed by our proposed model across all classes. For example, ResNet50V2 and DenseNet121, two of the stronger-performing baselines, achieved accuracy scores of 92%, which was notably lower than our proposed model’s 97%. The higher performance of our proposed model can be attributed to the addition of a channel-wise attention-based module, which enhances feature extraction by focusing on the most informative aspects of each input, thereby improving classification accuracy across complex scenarios involving fire, smoke, and neutral scenes. This comparison highlights the robustness of our proposed model in handling the complexities of multi-class fire and smoke detection. By leveraging the RGB color scheme and employing advanced feature refinement techniques, our model consistently outperformed established pretrained models, making it a promising solution for real-world fire detection applications that require high reliability and precision.

### 4.2. Comparison with State-of-the-Art Methods

We compare the performance of our proposed method against several state-of-the-art (SOTA) fire detection approaches, including FireXplainer [17], FireXplainNet [35], and FireDetXplainer [36]. Although these methods have achieved competitive results in previous studies, they were originally trained and evaluated on different datasets or configurations. To ensure a fair and consistent comparison, we re-implemented their architectures based on the descriptions provided in their respective papers, as the official code implementations are not publicly available. We then trained and tested these replicated models on our Fire-Smoke-Dataset using the same training, validation, and testing splits, as well as hyperparameter settings closely matching those described in their publications.

Table 1 presents the quantitative results of our comparison. The newly added SOTA methods—FireXplainer, FireXplainNet, and FireDetXplainer—demonstrate strong performance in terms of Accuracy, Precision, Recall, and F1-score. Specifically, FireXplainer attained an F1-score of 0.91, FireXplainNet reached 0.90, and FireDetXplainer achieved 0.92. Nevertheless, our proposed method outperforms all compared approaches, with an F1-score of 0.95. This improvement is also consistent across other metrics, indicating the robustness and generalizability of our framework.

It is important to highlight that minor discrepancies in performance might arise from inevitable implementation nuances, since we relied on the textual descriptions of the architectures and hyperparameters for replication. However, the replication effort provides a controlled setup where all methods are trained under identical conditions. Consequently, the results in Table 1 indicate that our method offers a superior balance of Accuracy, Precision, Recall, and F1-score for fire and smoke detection.

### 4.3. Impact of Augmentation on Fire Detection

To enhance the generalization capabilities of our proposed model, we employed a range of data augmentation techniques during training. Data augmentation is critical in deep learning, especially for fire detection tasks, where model robustness depends on exposure to diverse training examples, such as varying fire intensity, environmental conditions, and different types of smoke and fire behavior. In our experiments, we utilized augmentation methods such as random rotations, flips, scaling, and brightness adjustments, which artificially increased the variability of the training set. These augmented versions of images are included as part of the training set only, ensuring that the model is exposed to a wider range of scenarios during training while keeping the validation and test sets unaffected. The goal is to ensure that our model can effectively generalize across various real-world conditions, including different lighting scenarios, camera perspectives, and environmental obstructions.

The impact of data augmentation on model performance is summarized in Table 2. Overall, augmentation led to notable improvements in precision, recall, and F1-scores across all classes, contributing to a balanced and high-level performance. Specifically, it helped the model handle diverse scenarios more effectively by increasing variability in the training data. Specifically, the model achieved a precision of 96%, 98%, and 97% for the Fire, Neutral, and Smoke classes, respectively. Recall values are similarly high, with 96% for Fire, 99% for Neutral, and 96% for Smoke. The F1-score for each class are also consistent, with 96% for Fire and Smoke, and 99% for Neutral. The macro-averaged precision, recall, and F1-core are each 97%, indicating a balanced and high-level performance across all classes. The overall accuracy of the model is 97%, demonstrating the effectiveness of data augmentation in enhancing classification reliability.

The consistent precision and recall across all classes highlight the model’s robustness, particularly for challenging scenarios such as distinguishing smoke from background elements or detecting fire under varying environmental conditions. For instance, the model’s recall of 99% for the Neutral class suggests that it is highly effective in correctly identifying non-fire images, thereby reducing the likelihood of false positives. Similarly, the high precision and recall for the Fire and Smoke classes indicate that the model can accurately detect fire while minimizing false alarms and missed detections.

### 4.4. Impact of Color Schemes on Fire Detection

The evaluation of the our proposed model across various color schemes provides significant insights, revealing that the RGB scheme offers the highest performance, achieving an accuracy of 95%, as shown in Table 3. In addition to accuracy, the RGB color scheme also demonstrated superior precision (95%), recall (95%), and F1-score (95%), clearly outperforming other color schemes. This result underscores the importance of leveraging the full spectrum of color information available in the RGB format for effectively distinguishing between different classes in fire detection. The intricate variations in fire, influenced by factors such as material, lighting, and heat, are best represented in RGB, as it captures subtle color gradients, specific hues, and intensity variations that are crucial for accurate fire detection, contributing to its superior detection accuracy.

**Figure 3:**
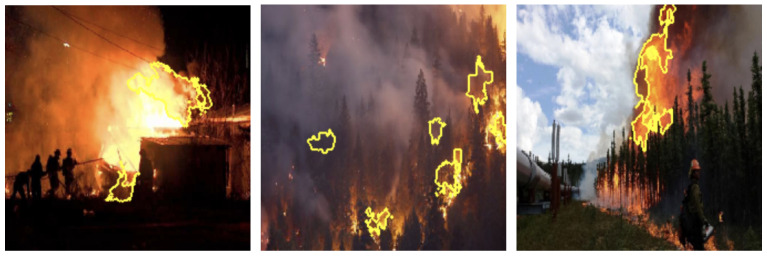
LIME visualizations in the RGB color space highlighting regions with the highest influence on fire detection predictions in representative images.

**Figure 4:**
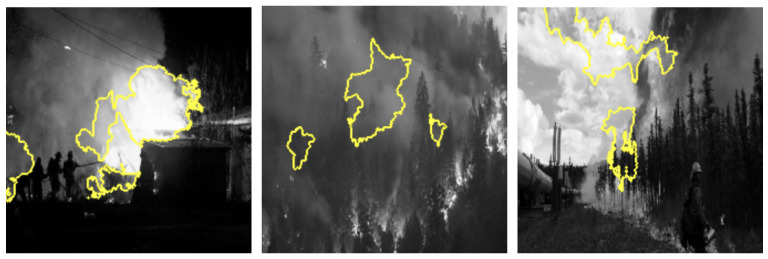
LIME visualizations in the Grayscale color space highlighting regions with the highest influence on fire detection predictions in representative images.

**Figure 5:**
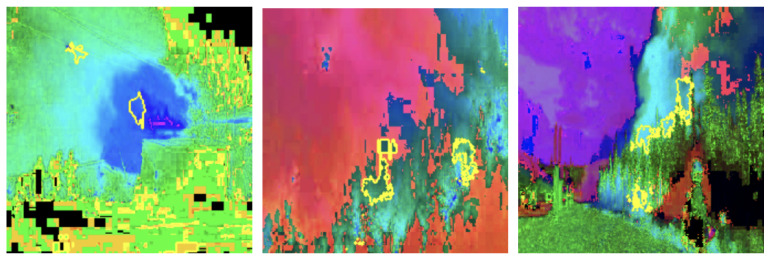
LIME visualizations in the HSV color space highlighting regions with the highest influence on fire detection predictions in representative images.

**Figure 6:**
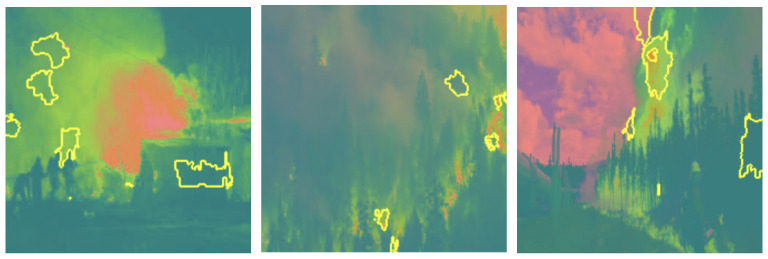
LIME visualizations in the YCbCr color space highlighting regions with the highest influence on fire detection predictions in representative images.

Grayscale and HSV color schemes yielded competitive results in fire detection; however, their performance metrics, such as accuracy and recall, are consistently lower compared to RGB, which demonstrated a stronger capability in distinguishing flame features from background elements. Specifically, Grayscale achieved an accuracy of 91%, precision of 91%, recall of 93%, and an F1-score of 92%. The absence of color information in Grayscale limits its ability to capture critical color cues, leading to reduced precision in certain scenarios. This indicates that intensity information alone, while important, is insufficient for robust fire detection. The HSV color scheme, on the other hand, achieved an accuracy of 92%, precision of 90%, recall of 94%, and an F1-score of 92%. While effective in capturing hue and saturation, HSV struggled with subtle distinctions in intensity that are better captured by RGB (as shown in Figure 3), resulting in marginally lower performance. LIME visualizations for Grayscale, HSV, and RGB, as shown in Figure 4, Figure 5, and Figure 6, respectively, further illustrate these observations. The LIME explanations demonstrate that the RGB color scheme provides a clearer distinction between flame and background regions, which supports its superior performance. These findings highlight that the additional color cues provided by RGB are essential for nuanced classification across fire, smoke, and natural scenes. The RGB color scheme, being the highest performing, are used for further evaluation and comparison with well-established pretrained models to ensure consistency and reliability in assessing model effectiveness.

### 4.5. Adversarial Attack Vulnerability

The Fast Gradient Sign Method (FGSM) [28] was employed to systematically assess the model’s robustness to adversarial perturbations, revealing significant performance variations under controlled attack conditions. Table 4 summarizes the quantitative impact of adversarial attacks on model classification performance. The introduction of FGSM perturbations resulted in a substantial reduction of overall accuracy from 95% to 41%. Precision metrics demonstrated considerable variability across classes, with Fire class precision declining to 0.37, Non-fire class to 0.41, and Smoke class to 0.45. Correspondingly, recall values exhibited similar degradation, indicating compromised feature discrimination under adversarial conditions.

**Figure 7:**
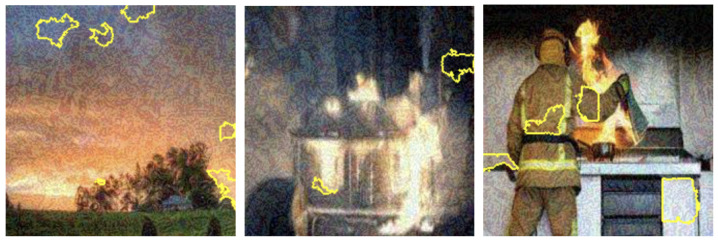
LIME saliency maps in RGB showing misclassifications from FGSM perturbations as focus shifts from flame regions.

**Figure 8:**
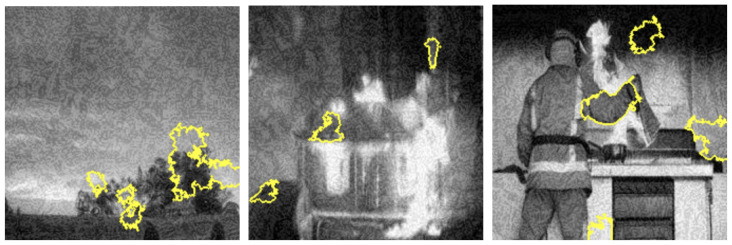
LIME saliency maps in Grayscale illustrating misclassification due to attention shifts under FGSM perturbations.

**Figure 9:**
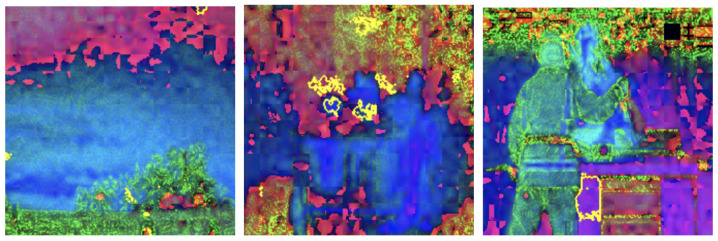
LIME saliency maps in HSV illustrating misclassification due to attention shifts under FGSM perturbations.

**Figure 10:**
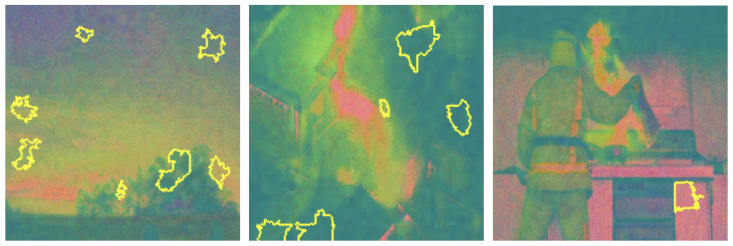
LIME saliency maps highlighting critical YCbCr regions influencing fire detection, showing misclassification patterns caused by FGSM-induced focus shifts.

LIME visualization techniques provided interpretable insights into the model’s decision-making processes across different color representations. Figure 7, Figure 8, Figure 9 and Figure 10 illustrate the shifts in feature attention induced by adversarial perturbations. Comparative analysis across color spaces revealed consistent patterns of feature attention disruption. In the original image space, LIME highlighted flame-specific regions with high specificity, while under FGSM perturbations, the saliency maps demonstrated significant redistribution of attention, with the model’s focus transitioning from flame-characteristic regions to peripheral image elements. The RGB color space exhibited the most pronounced misclassification patterns, with subtle input modifications substantially altering the model’s feature extraction mechanisms.

### 4.6. Model Explainability with LIME

We utilized LIME to evaluate the interpretability of our fire detection model by analyzing both original and adversarially attacked images. This approach allowed us to visually understand which features are most influential in the model’s predictions under different conditions.

To thoroughly assess the model’s behavior, we generated LIME visualizations for both original and adversarial images across various color schemes (e.g., RGB, Grayscale, HSV, and YCbCr). For the original images, LIME effectively highlighted regions corresponding to flame-specific features, especially in the RGB color scheme, as shown in Figure 3, Figure 4, Figure 5 and Figure 6. The visualizations showed that the model primarily focused on areas with intense red and orange hues, which are characteristic of flames. This indicates that the model can reliably localize relevant fire features in the RGB space, ensuring high interpretability. The LIME visualizations in Figure 3 demonstrate our model’s capability to simultaneously detect both fire and smoke features. In the first image, while the model identifies intense flame regions (shown by yellow highlights near the base), it also captures the rising smoke patterns above the fire. The second image shows how the model distinguishes between active flame zones and surrounding smoke diffusion patterns, with attention regions appropriately weighted based on feature intensity. The third image particularly highlights the model’s ability to differentiate smoke’s diffuse characteristics from the more concentrated fire regions, showing how channel-wise attention helps maintain distinct feature identification even when fire and smoke coexist in complex scenes. However, the Grayscale scheme, as well as other color schemes like HSV and YCbCr, produced broader and less distinct highlighted regions, demonstrating limitations in capturing the nuanced details that the model used to make predictions. This inconsistency in the identification of flame-specific areas across different color schemes suggests that RGB provides a more robust representation for localizing fire features. For adversarially perturbed images (using FGSM), LIME visualizations revealed significant changes in the model’s focus, as shown in the Figure 7, Figure 8, Figure 9 and Figure 10. The highlighted regions often shifted to irrelevant areas, such as background objects or non-flame regions, particularly in the RGB and HSV color spaces. This misdirection indicates the model’s susceptibility to adversarial noise, as it failed to consistently identify key features indicative of fire. The Grayscale and YCbCr visualizations exhibited similar disruptions, with less consistent and more diffuse areas of importance compared to original images.

The visualizations provide a comparative view of the model’s interpretability under original and adversarial conditions. In RGB, the saliency maps for original images are clear and focused on flame regions, supporting accurate predictions. Under adversarial conditions, however, the saliency maps became dispersed and misleading, demonstrating reduced reliability. HSV and YCbCr provided moderate results, capturing some important features but lacking the precision observed in RGB.

These findings emphasize the need for enhancing the model’s robustness against adversarial attacks. The visual evidence provided by LIME demonstrates that although the model performs well on original data, its reliability significantly diminishes under adversarial perturbations. This emphasizes the critical need for enhancing the model’s robustness against such attacks.

### 4.7. Ablation Study

To validate the effectiveness of our proposed channel-wise attention mechanism in fire detection, we conducted comprehensive ablation experiments. As each channel of a feature map is considered as a feature detector [46], channel-wise attention focuses on ’what’ is meaningful given an input image. We follow similar principles described in attention literature where channel relationships are explicitly modeled to enhance feature representation.

As shown in Table 5, the channel-wise attention mechanism provides substantial improvements over the baseline architecture. While the baseline MobileNetV2 achieves 0.92 accuracy, our channel-wise attention module enhances performance to 0.95 accuracy by adaptively recalibrating channel-wise feature responses. The attention mechanism helps the model focus on informative feature channels while suppressing less useful ones, leading to more effective feature refinement.

The performance improvement is consistent across all metrics, with both precision and recall increasing to 0.95, indicating that our channel-wise attention mechanism effectively helps the network concentrate on meaningful fire-specific features. This balanced improvement demonstrates that the feature refinement through channel-wise attention not only helps identify more fire instances correctly but also reduces false positives, which is crucial for reliable fire detection systems. These ablation results validate our architectural choice and demonstrate that explicitly modeling interdependencies between channels through attention improves the fire detection performance while maintaining computational efficiency.

## 5. Discussion

**Figure 11:**
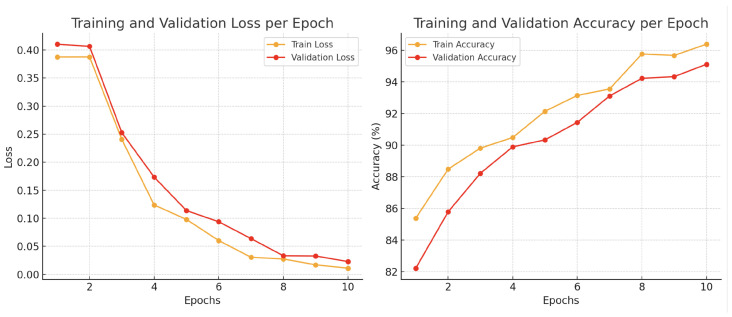
Training and validation accuracy and loss over 10 epochs.

Our comprehensive analysis of fire detection using a channel-wise attention-based MobileNetV2 architecture revealed critical insights into model performance, generalization, and robustness. The training dynamics, illustrated in the Figure 11, provide a nuanced view of our model’s learning progression and its ability to generalize across diverse fire detection scenarios. The training accuracy steadily increased from 82% at the first epoch to 95% by the tenth epoch, while the validation accuracy followed a similar trend, reaching 94% by the end of training. The close alignment between training and validation accuracy curves, both progressively improving and stabilizing around high values, suggests strong generalization to unseen data without signs of overfitting. Avoiding overfitting is particularly important for real-world fire detection scenarios, where the model must maintain high performance across a variety of environments and conditions that differ from the training data. Additionally, the training and validation loss decreased consistently, with the training loss dropping from 0.40 to 0.05 and the validation loss reducing from 0.42 to 0.06 over the ten epochs. The minimal divergence between the training and validation loss curves supports the robustness of the model, as both losses remained consistently low and converged smoothly. These findings collectively demonstrate that our proposed model maintains high predictive performance across different datasets and color schemes, making it a reliable solution for real-world fire detection applications.

Data augmentation emerged as a critical strategy for enhancing model generalizability. Our experiments demonstrated that techniques including rotations, scaling, and brightness adjustments increased overall accuracy by 7%, effectively mitigating overfitting in challenging scenarios with occlusions and varied lighting conditions. The augmentation approach addresses fundamental challenges in fire detection by artificially expanding the training data’s variability. This enables the model to adapt more effectively to real-world environmental complexities, particularly in scenarios with diverse fire intensities, smoke patterns, and background conditions. Comparative analysis with baseline models, such as VGG16, ResNet50V2, and EfficientNetB7, validated the efficacy of our augmentation strategy. Our attention-augmented MobileNetV2 outperformed these architectures by up to 8% in accuracy, highlighting the potential of sophisticated data augmentation techniques (as shown in the Table 2).

The comprehensive evaluation across different color spaces revealed critical insights into feature representation. The RGB color scheme consistently outperformed alternative representations, achieving 95% accuracy compared to other color spaces’ 90–92% performance. This superiority stems from RGB’s ability to capture comprehensive color and intensity variations crucial for distinguishing flame characteristics. Our analysis showed that while Grayscale and HSV schemes provided competitive results, they struggled to capture the nuanced visual cues essential for accurate fire detection. The RGB scheme’s ability to preserve subtle color gradients and intensity variations proved instrumental in differentiating fire from complex backgrounds. LIME visualizations further substantiated these findings, demonstrating that the RGB color space provides the clearest distinction between flame and non-flame regions, underscoring the importance of color information in developing robust fire detection models (as shown in the Figure 3, Figure 4, Figure 5 and Figure 6). The ablation study results further validate our architectural choices, demonstrating that the channel-wise attention mechanism contributes significantly to the model’s performance. The 3% improvement in accuracy from adding channel-wise attention confirms the mechanism’s effectiveness in focusing on flame-specific and smoke features while suppressing background noise.

The investigation into adversarial vulnerabilities revealed significant challenges in deploying deep learning models for safety-critical fire detection applications. FGSM attacks demonstrated a dramatic performance reduction from 95% to 41% accuracy, highlighting the model’s susceptibility to minimal input perturbations (as shown in the Table 4). LIME visualizations provided critical insights into these vulnerabilities, showing how adversarial attacks can fundamentally alter the model’s feature attention mechanisms (as depicted in the Figure 7, Figure 8, Figure 9 and Figure 10). Under perturbation, the model’s focus shifted from flame-specific regions to irrelevant background elements, exposing potential weaknesses in current deep learning approaches. This analysis underscores the need for developing more robust detection systems capable of maintaining consistent performance under diverse and potentially manipulated input conditions. The findings contribute to the broader research discourse on adversarial resilience in computer vision applications.

The interconnected insights from training dynamics, augmentation, color space analysis, and adversarial vulnerability assessment highlight the complex challenges in developing reliable fire detection systems. Our approach demonstrates that addressing these challenges requires a multifaceted strategy involving sophisticated feature extraction, comprehensive training techniques, and rigorous robustness evaluation. The channel-wise attention mechanism, combined with careful data augmentation and color space selection, provides a promising pathway for enhancing fire detection model performance and reliability.

## 6. Conclusions

This study comprehensively evaluates model interpretability and adversarial vulnerabilities in fire detection through channel-wise attention-based MobileNetV2 architecture. Our systematic analysis yielded several key insights: (i) Channel-wise attention mechanisms significantly enhance feature discrimination, enabling the model to effectively filter background noise while maintaining focus on flame-relevant regions; (ii) Data augmentation techniques substantially improve model generalization, achieving 97% accuracy across precision, recall, and F1-score metrics by exposing the model to diverse environmental conditions; (iii) The RGB color scheme provides optimal performance with 95% accuracy, demonstrating the importance of comprehensive color information for distinguishing flame-specific features.

Our analysis of adversarial vulnerabilities revealed critical security considerations for deploying deep learning models in fire detection systems. FGSM perturbations caused significant performance degradation, reducing accuracy to 41%. LIME visualizations demonstrated how adversarial attacks shift the model’s attention from flame regions to irrelevant background elements, highlighting the need for enhanced robustness in safety-critical applications. These findings underscore the importance of comprehensive security evaluation before deploying such systems in real-world scenarios.

Future work will focus on enhancing model resilience to adversarial attacks through domain-adaptive training methods, such as Bilateral Adversarial Training (BAT) [50], to ensure robust performance across varying noise levels and environmental conditions. Additionally, we plan to investigate advanced Vision-Language Models (VLMs), including CLIP [51] for improved image-text alignment, Flamingo [52] for few-shot adaptation in diverse environments, and multimodal capabilities of GPT-4 [53] and LLaMA 3.2 [54] to enhance real-time scene understanding and context-aware detection. These models, known for their success in medical diagnostics [55] and autonomous driving [56], can allow fire detection systems to better discern flame characteristics amidst complex backgrounds. For augmentation, we will incorporate techniques including Gaussian noise injection [57], elastic transformations [58], blur, and color jittering [59] to replicate challenging real-world conditions, such as smoke interference, variable fire intensities, and adverse weather.

## Figures and Tables

**Table 1:** Multi-Class Performance Metrics for Fire, Smoke, and Non-Fire Detection Using the Fire-Smoke-Dataset.

Model	Accuracy (%)	Precision (%)	Recall (%)	F1-Score (%)
VGG16 [24]	0.78	0.78	0.77	0.78
VGG19 [24]	0.80	0.82	0.79	0.81
MobileNetv2 [19]	0.92	0.90	0.93	0.92
EfficientNetB7 [32]	0.82	0.82	0.81	0.81
ResNet50v2 [25]	0.92	0.92	0.91	0.91
Xception [14]	0.91	0.91	0.91	0.91
DenseNet121 [33]	0.92	0.93	0.94	0.93
InceptionV3 [34]	0.92	0.92	0.92	0.92
FireXplainer [17]	0.92	0.91	0.91	0.91
FireXplainNet [35]	0.90	0.90	0.89	0.90
FireDetXplainer [36]	0.91	0.92	0.92	0.92
Ours	0.95	0.95	0.95	0.95

**Table 2:** Impact of augmentation of trained images on fire detection and classification.

Class	Precision	Recall	F1-Score	Accuracy
Fire	0.96	0.96	0.96	0.97
Neutral	0.98	0.99	0.99	0.97
Smoke	0.97	0.96	0.96	0.97
Macro avg	0.97	0.97	0.97	0.97
Weighted avg	0.97	0.97	0.97	0.97

**Table 3:** Comparation of our approach for fire detection using different color schemes.

Color Scheme	Accuracy (%)	Precision (%)	Recall (%)	F1-Score (%)
Grayscale	0.91	0.91	0.93	0.92
HSV	0.92	0.90	0.94	0.92
Ycbcr	0.91	0.92	0.90	0.91
Red	0.90	0.90	0.90	0.90
Blue	0.92	0.89	0.93	0.92
Green	0.92	0.90	0.90	0.90
RGB	0.95	0.95	0.95	0.95

**Table 4:** Impact of adversarial attacks on fire detection and classification.

Class	Precision	Recall	F1-Score	Accuracy
Fire	0.37	0.25	0.30	0.41
Non-fire	0.41	0.77	0.54	0.41
Smoke	0.45	0.21	0.29	0.41
Macro avg	0.41	0.41	0.38	0.41
Weighted avg	0.41	0.41	0.38	0.41

**Table 5:** Impact of channel-wise attention mechanism on fire detection performance.

Model	Accuracy	Precision	Recall	F1-Score
MobileNetV2	0.92	0.90	0.93	0.92
MobileNetV2 + Attention	0.95	0.95	0.95	0.95

## Data Availability

The datasets utilized in this study include the Fire-Smoke-Dataset curated by DeepQuestAI, accessible on GitHub at https://github.com/DeepQuestAI/Fire-Smoke-Dataset (accessed on 1 September 2024).

## Abbreviations

The following abbreviations are used in this manuscript:

CNN	Convolutional Neural Network
MLP	Multi-Layer Perceptron
LIME	Local Interpretable Model-Agnostic Explanations
FGSM	Fast Gradient Sign Method
PGD	Projected Gradient Descent
VLM	Vision-Language Model
RGB	Red Green Blue
HSV	Hue Saturation Value
YCbCr	Luminance Chrominance
BAT	Bilateral Adversarial Training
